# Supplementary material for: The Free Radical Scavenging Property of the Leaves, Branches, and Roots of Mansoa hirsuta DC: In Vitro Assessment, 3D Pharmacophore, and Molecular Docking Study
Source: Molecules. 2022 Sep 15;27(18):6016. doi: 10.3390/molecules27186016 (PMC9506257; doi:10.3390/molecules27186016)
Supplement: Supplementary file 1 [file molecules-27-06016-s001.zip › molecules-1920018-supplementary.pdf]

molecules

MDPI

Supplementary Material

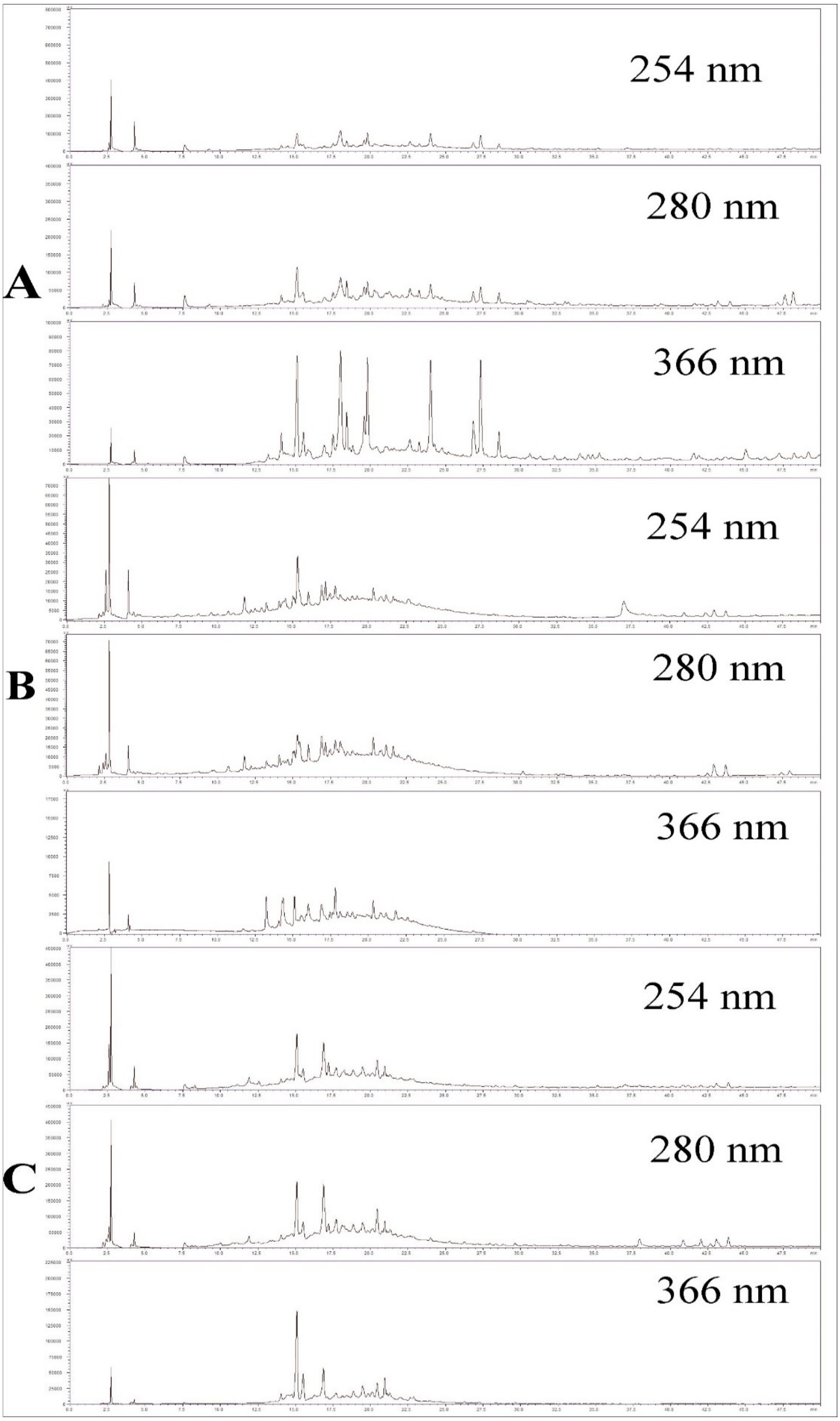

a: leaves; b: branches; c: roots.

Figure S1. Analytical chromatograms of methanol extract of *M. hirsuta* by HPLC-PDA, gradient mode in 65.5 min, injected sample solution 1 mg. mL^{-1}, flow 1 mL. min^{-1}, C_{18} reverse phase column, detection at wavelengths 254 nm, 280 nm and 366 nm, [a] leaves, [b] branches and [c] roots

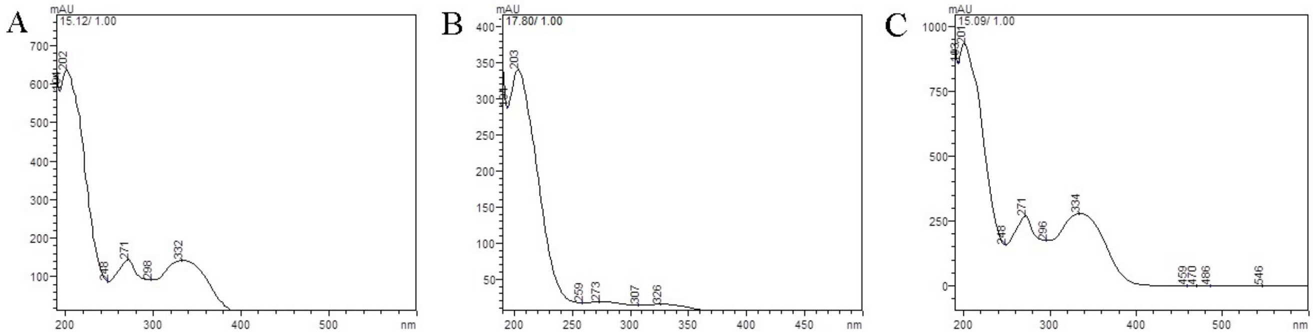

a: leaves; b: branches; c: roots.

Figure S2. UV spectra at wavelengths in the range of 200 nm to 334 nm at the retention time 16.12min, 17.80min and 15.05min respectively. [a] leaves, [b] branches and [c] roots

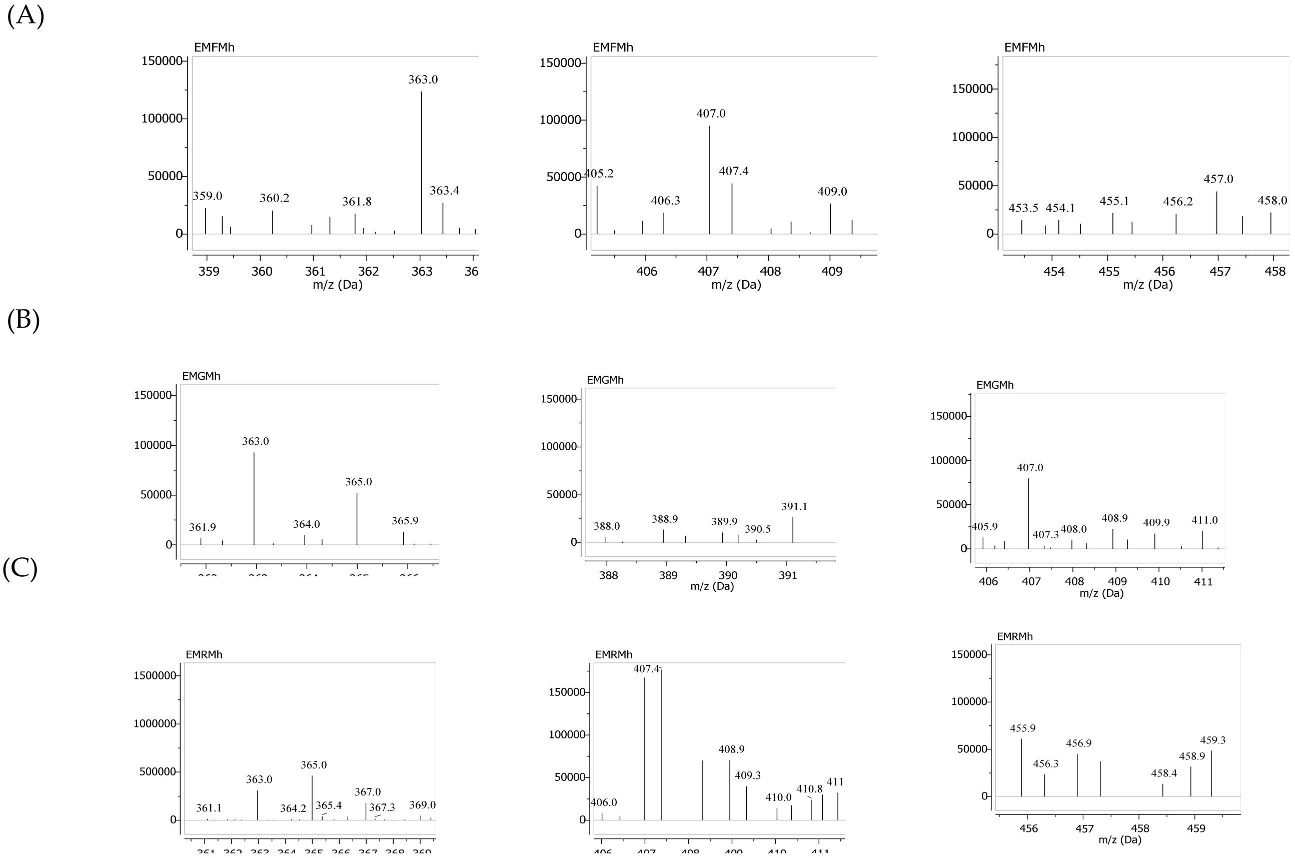

Figure S3. Spectra (A) leaves (B) branches (3) and (C) roots with the corresponding *m/z* fragments in the negative ionization mode of oleonolic and ursolic acid

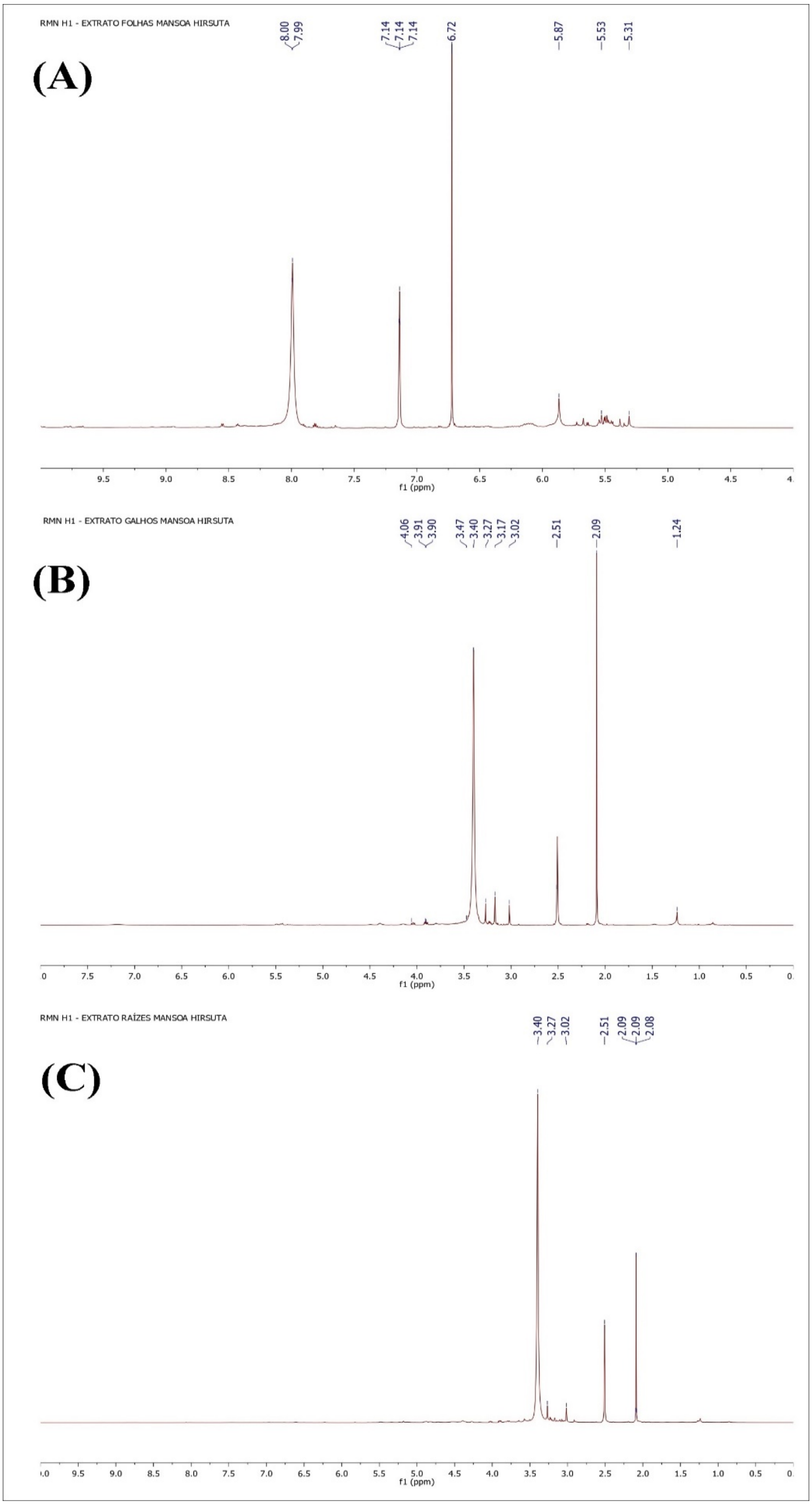

a: leaves; b: branches; c: roots.

Figure S4. ^{1}H NMR spectra (DMSO-d6, 600 MHz) of methanol extract of leaves, branches and roots of *M. hirsuta*

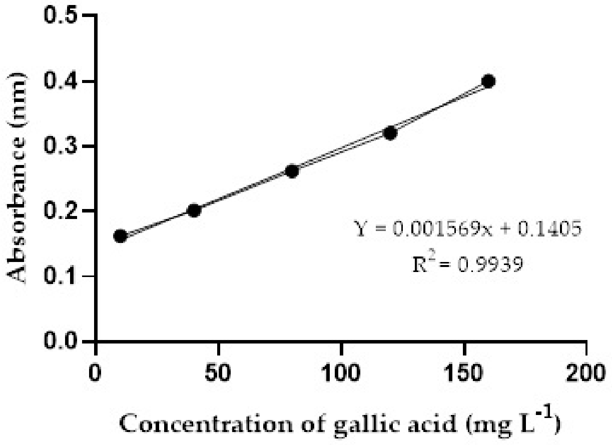

Figure S5. Standard curve of total phenols from *M. hirsuta* extracts
